# The Improved Measurement of Building Thermal Transmittance in Zagreb Using a Temperature-Based Method

**DOI:** 10.3390/s25113456

**Published:** 2025-05-30

**Authors:** Igor Štambuk, Roman Malarić, Ivica Bakota, Zvonko Trzun

**Affiliations:** 1 University of Defense and Security, 10000 Zagreb, Croatia; istambuk.edu@gmail.com (I.Š.); zvonko.trzun@gmail.com (Z.T.); 2 Faculty of Electrical Engineering and Computing, University of Zagreb, 10000 Zagreb, Croatia; 3 VIB Buildings, 21300 Makarska, Croatia; ivicabakota@yahoo.com

**Keywords:** thermal transmittance, heat flow meter, opaque element, buildings

## Abstract

Theoretical *U*-values, which measure thermal transmittance, can be calculated based on the thermal parameters of an opaque element’s layers. However, practical measurements are essential to validate these theoretical values. The heat flux meter (HFM) method, is a widely accepted standard for such measurements. Despite its prevalence, the HFM method faces challenges, including wall surface roughness, ensuring proper contact between measurement devices and surfaces, and weather-related fluctuations. This study introduces a prototype system that employs a modified temperature-based method (TBM) to address these challenges. The paper provides a detailed comparison of thermal transmittance measurements obtained using both the modified TBM and the HFM method. The results showed *U*-value differences between the two methods. Additionally, these experimental findings were compared with theoretical calculations, highlighting the efficacy and potential of the modified TBM as an alternative approach for accurate *U*-value determination.

## 1. Introduction

Energy efficiency has emerged as a critical concern in today’s world, capturing the attention of scientists and engineers across various disciplines. From physics to electrical, mechanical, chemical, civil, and traffic engineering, professionals are deeply invested in addressing the environmental and economic impacts of reducing energy consumption in daily life. With advancing technology and rising living standards, heating and cooling systems have become significant energy consumers. This highlights the urgent need to tackle heat loss through building envelopes. The thermal properties of walls play a pivotal role in assessing insulation quality, which can deteriorate over time. Accurately identifying these properties presents a considerable challenge. This paper examines a method that is particularly advantageous in cases where efforts are being made to improve the thermal insulation of numerous old buildings that were constructed without adequate thermal protection. For cases involving large-scale facilities (e.g., hospitals, university campuses, military bases, etc.), engineers and researchers strive to obtain reliable data without the need for expensive heat flux sensors, making it an attractive alternative to conventional measurement techniques.

The energy performance of a building envelope, including its *U*-values, can be analytically determined if the material properties of its layers and environmental influences are well-documented [1,2,3,4,5]. However, in practice, such precise data are often unavailable or compromised by aging and other factors. Consequently, there is an increasing demand for accurate *U*-value data derived from empirical in situ measurements, enabling experts to make informed assessments and improvements.

As part of the growing interest in accurately determining the thermal transmittance of building elements, several parallel studies have been conducted with the aim of developing methods that enable simple, fast, and accurate *U*-value measurements. An experimental setup developed in the laboratory of Roma TRE University uses a low-cost device for measuring heat flux through a thermometric method, demonstrating that even with limited resources, reliable results can be achieved [6]. Another study emphasizes the importance of characterizing the thermal properties of building envelopes in the context of the EU’s 2050 climate goals and showed that by using thermometric variables alone—without direct heat flux measurements—thermal transmittance can be estimated with high accuracy [7]. An alternative *U*-value measurement method has been developed based solely on the measurement of three temperatures (indoor air, external wall surface, and inside the wall), eliminating the need for heat flux sensors. This method stands out for its simplicity and low cost [8]. Some authors reviewed the THM, which, despite its simplicity, showed better performance during winter months. They concluded that in Mediterranean climate conditions, results are less reliable without a temperature difference of at least 5 °C, while a difference greater than 10 °C significantly improves measurement accuracy [9]. Regarding the TBM, some progress has been made in recent years. In the literature, the method is also called the thermometric method (THM) or the air-to-surface temperature ratio (ASTR) method. It enables precise short-term measurements with a low error margin (±3%) compared to long-term HFM measurements. This method has proven particularly useful for post-analysis of insulation effects [10]. The ASTR method has been further improved by focusing on the importance of achieving quasi-steady-state conditions during measurements. It has been shown that measurement uncertainty increases significantly if such conditions are not met, while sampling under stable conditions greatly improves accuracy. Furthermore, an additional correction of the heat transfer coefficient is proposed for buildings with underfloor heating, enhancing the reliability of measurements in practical applications [11].

Measuring the energy envelopes of buildings is important for saving electricity, working climate and a pleasant feeling. Since the C building of FER is currently being renovated after the earthquake, the topic is interesting to us, and several papers have been published about it [12,13]. In addition to the above, another motivation is to develop a system that would be better than the existing commercial measurement systems in terms of precision and capabilities.

In relation to previous research work [12,13,14], a system for measurements using the two methods, HTM and TBF, was designed, the measurement process was automated, and data processing was improved using the LabVIEW program. By special selection and calibration of measuring thermistors, lower measurement uncertainty was achieved. Thermal conductivity calculated by the two methods was compared and deviations were determined. Measurement results were also compared with theoretically calculated values.

There are two primary methods available for *U*-value measurement: the temperature-based method (TBM) and the heat flux meter (HFM) method. Both provide in situ *U*-values, but they differ significantly in quality and accuracy. The distinction lies in how the *U*-value is calculated, as shown by the following formula:

(1) $$U\;\left(\frac{W}{m^{2}K}\right)=\frac{Q}{T_{in}-T_{out}}$$

Here, *T_in_* and *T_out_* represent indoor and outdoor temperatures (Kelvin), and *Q* is the heat flux through the wall (W/m^2^). Both methods rely on sensors to measure *T_in_* and *T_out_*, but the approach to determining *Q* differs. In the HFM method, *Q* is directly measured using a heat flux sensor placed on the inner wall surface, while *T_in_* and *T_out_* are recorded by temperature sensors. This method ensures all parameters for *U*-value calculation are directly obtained. The TBM; however, approximates *Q* by measuring *T*_in_ and the wall surface temperature (*T_wall_*), assuming a fixed thermal boundary resistance (*R_si_*) between the wall and indoor air:

(2) $$\;\;\;Q\;\left(\frac{W}{m^{2}}\right)=\frac{T_{in}-T_{wall}}{R_{si}}$$

Here, *T_wall_* is measured with a sensor, while *R_si_* is estimated using standard values for building materials, often 0.13 m^2^K/W [5]. As *R_si_* is an assumption rather than a direct measurement, it may differ substantially from actual conditions, affecting accuracy. *Q* was then used in the *U*-value formula with an additional sensor for *T_out_*. According to these considerations, many measurements were made [4,5,6,7,8,9,10,11,12,13,14,15,16,17,18,19,20,21,22]. Using this method, better sensitivity and accuracy were achieved because a 6.5-digit high-precision digital multimeter was used for the measurement. Furthermore, all temperature sensors were calibrated to ensure a precision of better than 1 mK between sensors. The structures of these results are well explained.

**Figure 1:**
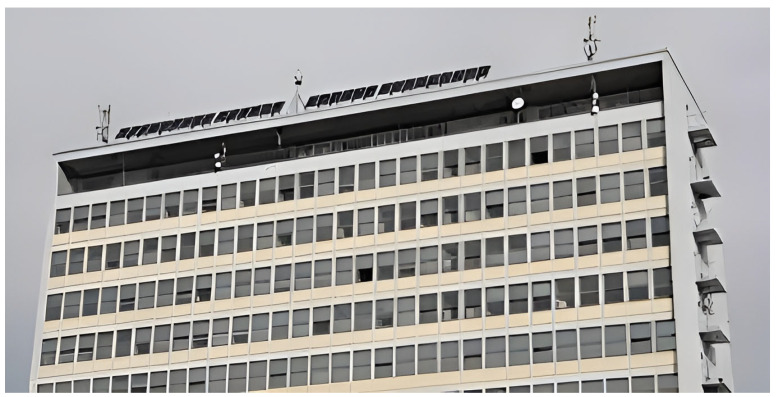
Faculty of Electrical Engineering Building in Zagreb (FER).

In this paper, a new system for measuring the *U*-value was designed and modified. Both the temperature-based method (TBM) and the heat flux meter (HFM) method were used in the measurements. In the first case, a wall at the Faculty of Electrical Engineering building in Zagreb (FER) was tested, and in the second, a window of the same building was tested. The building can be seen in Figure 1.

## 2. Measurement System Setup

The *U*-value is measured in two distinct locations. The first location is the external wall of the building, and the second is an external window on the same building as seen on Figure 1. The temperature is measured on both sides of the wall and the heat flux from the inside of the laboratory is measured. After that, the measured and simulated heat fluxes at the inner edge of the wall are compared and the simulated heat flux is iteratively calculated to obtain the thermal parameters of the wall. The wall studied in this experiment is located on the west side of the building, protected all day from direct sunlight by neighboring buildings. The wall is 37 cm thick and made of brick with an unknown thickness of insulation (a thickness of 5 cm was used in the calculation).

In this experiment, two heat flux sensors are used, and the temperature is measured by NTC thermistors EC95F103 placed near the heat flux sensor on the wall surface and on sensors located away from the wall to determine the temperature in the interior and exterior space. The room is maintained at an approximately constant temperature by means of an air conditioner. The measurement is performed during several cold winter days, from which a period of 200 h (9 days) of periodic temperature changes is analyzed and presented.

**Figure 2:**
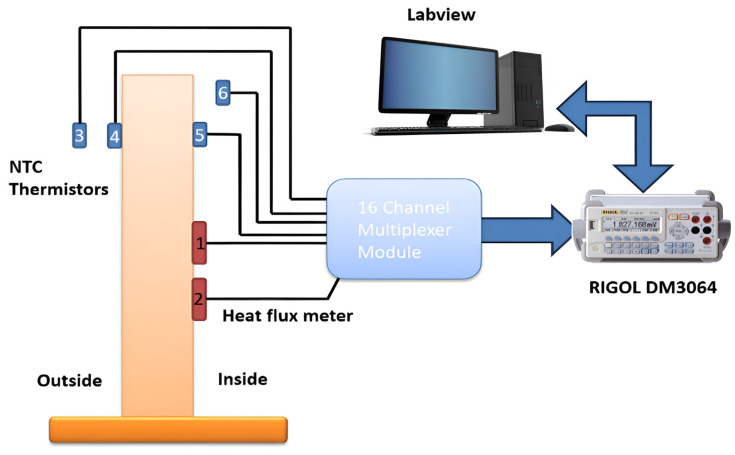
Measurement system diagram: 1. Huxeflux thermal heat flux plate HFP01; 2. Huxeflux thermal heat flux plate HFP01; 3. temperature sensor *T_out_*; 4. temperature sensor *T_wout_*; 5. temperature sensor *T_win_*; 6. temperature sensor *T_in_*.

The experimental setup is shown in Figure 2.

**Figure 3:**
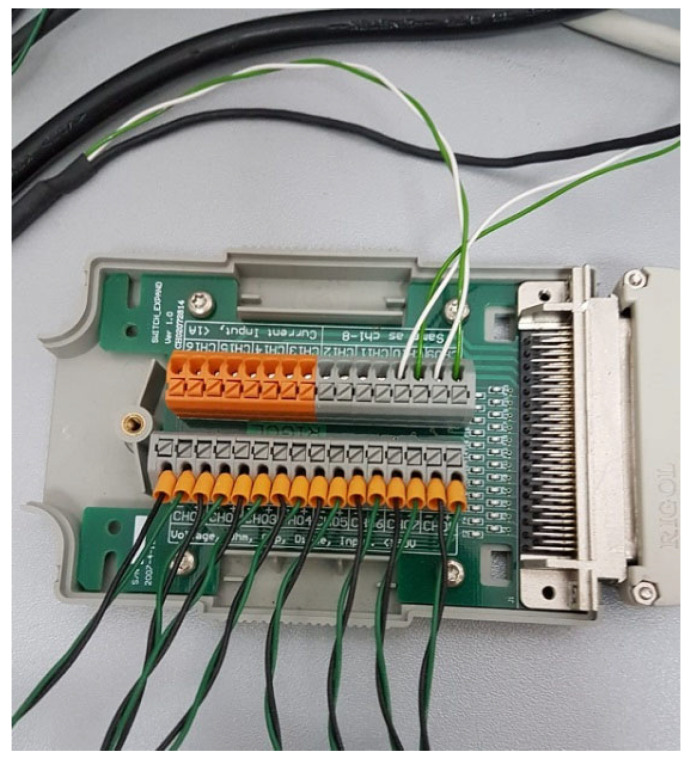
The 16-channel multiplexer module used in measurements.

Measurement system setup consists of a Huxeflux thermal heat flux plate HFP01 [23] as the heat flux sensor. The total thermal resistance is kept small by using a ceramic–plastic composite body, and the sensor is suitable for long term use. The measurement range is −2000 to +2000 W/m^2^, with sensitivity of 60 × 10^−6^ V/(W/m^2^). The low output voltage of such sensors makes it difficult to measure small voltages in the range of microvolts; however, the chosen digital multimeter has resolution of 0.1 µV on the voltage range of 200 mV and acceptable accuracy, so it was not necessary to consider nanovoltmeter or similar instruments. The advantage of this instrument is that it has a 16-channel multiplexer module (Figure 3), which can be adapted to measure both voltages and resistances.

The whole measurement system consists of two thermal flux sensors, four to ten NTC thermistors for averaging temperatures, a digital multimeter, and a computer for measurement and control. The LabVIEW programming environment is chosen to control the equipment and for data collection.

The NTC thermistor EC95F103V is a negative temperature coefficient device optimized for accurate temperature sensing and control. It has a nominal resistance of 10 kΩ at 25 °C, providing a highly sensitive thermal response over its specified operating range of 0 °C to 70 °C. The thermistor supports a maximum power dissipation of 75 mW, which minimizes self-heating and ensures measurement stability. Calibration of the thermistors was performed at the National Metrology Institute of Croatia, with a temperature uncertainty of 30 mK. Furthermore, all NTC thermistors used in this study were mutually calibrated, with inter-sensor deviations maintained below 1 mK, ensuring a high degree of measurement consistency.

The above measurement system was used to measure the *U*-value according to two methods, the temperature-based method (TBM) and the heat flux meter (HFM) method. The methodology differs in certain aspects, except for the measurement system, particularly in the case of assessments conducted using the modified TBM. The experimental setup consisted of laboratory rooms separated by a partition wall, which were actively conditioned (heated or cooled) throughout the entire measurement period to maintain stable and uniform temperatures. A minimum temperature differential of 10 °C between the wall surfaces was ensured [1]. Temperature readings were continuously monitored and processed using the LabVIEW 2015 software application.

The temperature measurement system developed is superior to existing commercial ones in terms of precision and capabilities, consisting of 16 measurement channels that can be used both for temperature measurement (resistance measurement) and for measuring the output voltage of the heat flux sensor. In this way, it is possible to place several temperature sensors in different parts of the wall (or other areas to be assessed) which can be used to obtain more reliable results. Using this method, better sensitivity and accuracies are achieved because a high-precision, 6.5-digit digital multimeter is used for measurement. Furthermore, all temperature sensors are calibrated, which ensures a precision between sensors better than 1 mK. The measurement method is programmed in the LabVIEW programming language, and the measurement period can be arbitrarily selected, even modified on the front panel of the measuring system. The period is selected considering the actual conditions, without downloading unnecessary data, but with the possibility of recording all transient changes in climatic conditions.

According to ISO 9869-1 standard [24], the duration of a thermal performance test is determined based on whether the measured values meet the following three criteria:

1. The test duration must exceed three days.
2. The R-value calculated at the end of the test must not differ by more than ±5% from the R-value obtained one day earlier.
3. The R-value derived from the data collected during the initial period of INT (2 × DT/3) days must not deviate by more than ±5% from the R-value calculated over the final period of the same length, where DT is the total duration of the test in days and INT denotes an integer.

The standard allows for measurement durations ranging from a minimum of 3 days to more than 7 days [25]. However, in practice, many studies have extended the measurement period to approximately one week, and in some cases, to more than two weeks, to ensure the reliability and stability of the results.

The results of this study are in accordance with that standard, and the measurement uncertainty of the measurement is far below 5%. This measuring system is very precise, far more precise than others on the market that offer one sensor and a measuring system of low accuracy. This system allows more channels, better precision, a large number of measurements, the possibility of changing the measurement parameters from sampling to the measurement period, etc.

### 2.1. Measurement Uncertainty in the HFM and TBM

The measurement uncertainty associated with the heat flux meter (HFM) method is predominantly governed by type B uncertainty, as specified in the instrument’s technical documentation. Type A uncertainty is negligible in comparison. Consequently, the overall measurement uncertainty, whether for wall or window measurements, by using this method, better sensitivity and accuracy are achieved because a 6.5-digit high-precision digital multimeter is used for the measurement. Furthermore, all temperature sensors are calibrated to ensure a precision of greater than 1 mK between sensors, which is approximately 1%, with a coverage factor of *k* = 1.

In the temperature-based method (TBM), the primary source of uncertainty is also type B, from the temperature sensor’s uncertainty of 30 mK. Additionally, the TBM’s accuracy is influenced by the temperature difference between the interior and the wall surface: smaller temperature differences lead to increased uncertainty. Therefore, the TBM generally exhibits higher uncertainty than the HFM method under low thermal gradients. However, at larger temperature differences, the TBM may yield lower measurement uncertainty than the HFM method.

**Figure 4:**
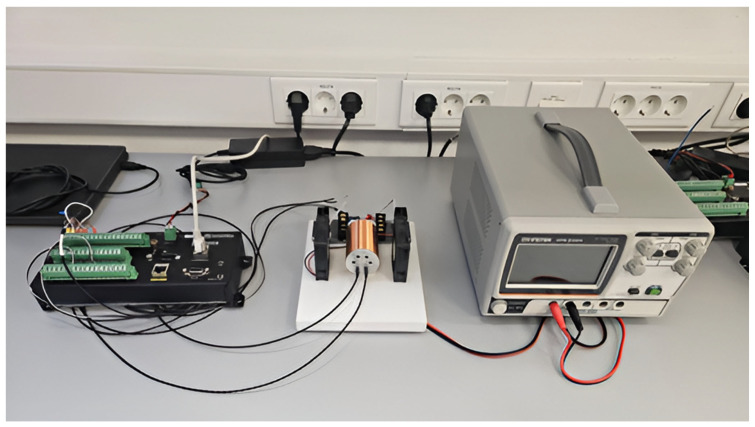
Comparison of NTC thermistors.

During calibration, all thermistors are mounted within a thermally stable aluminum enclosure. By varying the current through the surrounding coils, the internal temperature of the housing can be precisely controlled, enabling comparative analysis of different NTC thermistors. For each thermistor, the parameters of the nonlinear temperature–resistance relationship are calculated, ensuring consistent and accurate measurements across a broad temperature range, with a resolution of greater than 1 mK. See Figure 4.

### 2.2. Temperature and Meteorological Characteristics of the Measurement Site

Prior to sensor placement, thermographic imaging was employed to identify the optimal region of the wall surface for thermal conductivity measurements. Temperature probes and heat flux meter plates were then precisely positioned and securely affixed using self-adhesive tape. To minimize parasitic thermal resistance caused by inadequate contact, additional measures were taken to enhance the thermal interface between the temperature probes and the wall surface.

The measurement location, i.e., the building of the Faculty of Electrical Engineering and Computing, University of Zagreb, is in Zagreb, in the region of the City of Zagreb, in northwestern Croatia: the coordinates are 45°48′46″ N, 15°58′39″ E.

**Figure 5:**
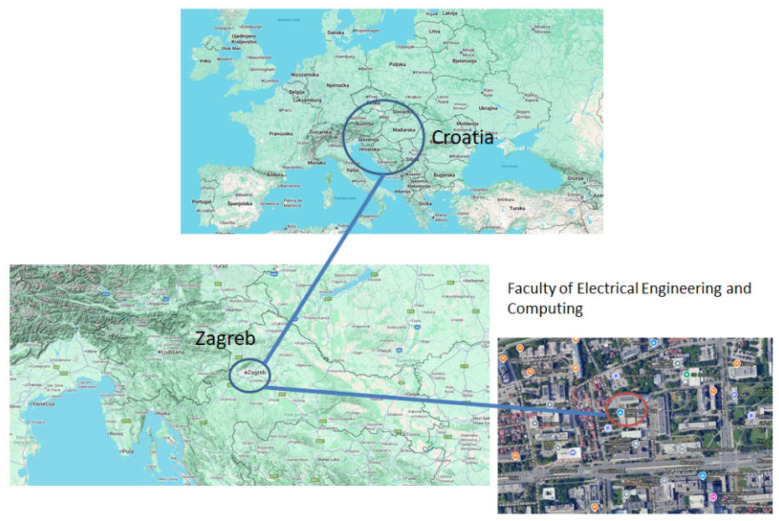
Map showing the location of Zagreb and the FER.

**Figure 6:**
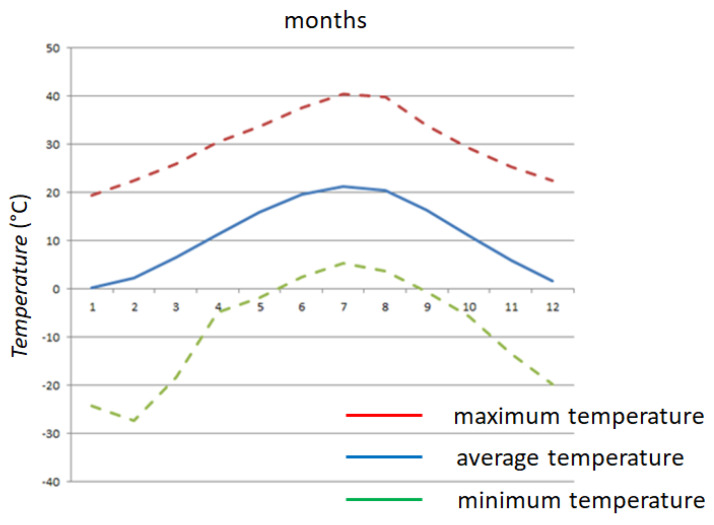
Average annual change in air temperature in Zagreb.

Figure 5 presents a map highlighting the location of Zagreb. Figure 5 illustrates the anticipated trends in air temperature throughout the year based on data recorded in Zagreb and sourced from the State Hydrometeorological Institute (DHMZ) archive [26]. In Figure 6, the months are numbered from 1 (January) to 12 (December). The graph features a continuous line to represent mean values, while dashed lines indicate the minimum and maximum expected values, offering a comprehensive view of seasonal variations.

The study focused on rooms located on the north side of the building, shielded from direct solar radiation by nearby structures. The rooms remained unoccupied and inaccessible to students throughout the testing period.

## 3. Analysis of Measurement Results

### 3.1. Measurement of U-Value of External Wall: Case A

For case A, a preliminary investigation was conducted using a Flir T620 thermal camera in accordance with the ISO 6946:2017 standard [27]. This initial assessment identified the most suitable area for thermal transmittance measurement using the heat flux meter (HFM) method. Infrared thermography was employed to inspect the wall surfaces, ensuring there were no thermal bridges, damage to the plaster, or moisture infiltration that could affect the opaque elements. The wall was tested for thermal uniformity and the presence of thermal bridges, to make it as suitable as possible for measurements.

**Figure 7:**
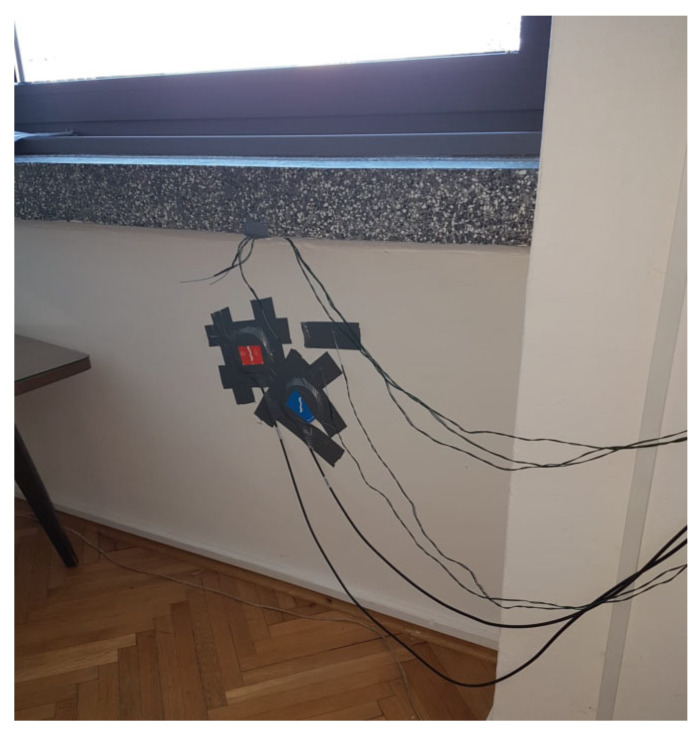
Measurement setup: two probes are attached to the wall.

The HFM method, as described in ISO 9869-1:2014 [1], is a widely accepted approach for determining the thermal conductivity of opaque elements [4]. This method involves monitoring temperature variations on both sides of the opaque element over time and measuring the heat flux passing through the wall. Standard practice includes attaching a heat flux measuring plate to the wall surface at a designated location to capture heat flow across the opaque element’s cross-section. Temperature probes are placed a few centimeters away from the plate and secured with adhesive tape. Two sensors are placed on the same side of the wall, so that the results of one sensor are positive and the other negative to eliminate any systematic errors, as seen in Figure 7 (blue and red colored panels). The final result for HFM sensors is an average of two sensors.

Our experiment also implemented a modified approach to the TBM, introducing adjustments aimed at improving the accuracy and reliability of measurements. The specifics of this modified method and the results obtained from the measurements are discussed further in the study. Measurements were also performed according to the HFM method, also considering the reliability of the measurements, and the results were compared.

To ensure uniform and stable temperatures, the rooms were air-conditioned, either heated or cooled, for 24 h, maintaining a temperature difference of no less than 10 °C between the inside and outside of the window or wall. The specific temperature values varied across tested cases and are detailed in Section 3. Thermographic examination was employed to identify the most suitable area of the wall surface for thermal transmittance measurement. The heat flux meter plate was positioned 0.5 m above the floor, with sensor placement carefully planned. Technicians used color-coded sensors to ensure accurate identification of their positions on the opaque wall. Sensor placement relative to the heat flux plate was precisely determined to facilitate multiple measurements across different areas of the wall. To attach the heat flux sensors to the opaque wall, adhesive tape was used for easy removal after the experiments. To improve contact between the temperature probes and the surface on which they are placed, thereby minimizing unwanted thermal resistance caused by poor contact, commercial high-temperature silicone grease was applied to the selected areas. Each temperature probe was then secured to the silicone within a specialized casing. The casing’s aluminum base, in contact with the wall, ensured optimal thermal conductivity, while the outer plastic layer provided durability and insulation.

After room conditioning, heat flux and temperatures were monitored and recorded for a duration of nine days. Once data collection was completed, an integral approach was applied to evaluate the thermal conductivity of the walls, providing a comprehensive understanding of their performance.

**Figure 8:**
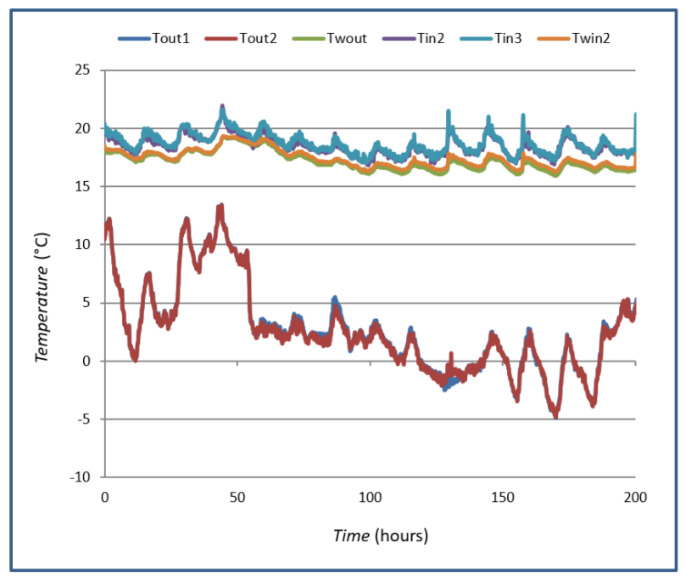
Measurement of thermal oscillations in case A.

For the case of measuring the *U*-value on the wall, continuous measurements of the wall temperature on both sides (*T_win_* and *T_wout_*), the air temperature at 10 cm from the wall (*T_in_* and *T_out_*), and the heat flow through the wall *Q* were carried out. For case A, the measurements were carried out in January, and the room where the measurements were carried out was conditioned at *T* = 20 °C to ensure a temperature difference greater than 10 °C according to the requirements of the standard ISO 9869-1:2014 [1]. Figure 8 shows the trends of the registered temperatures. The temperatures recorded are external temperature, external wall temperature, internal temperature and internal wall temperature. Measurements are carried out with several sensors.

**Figure 9:**
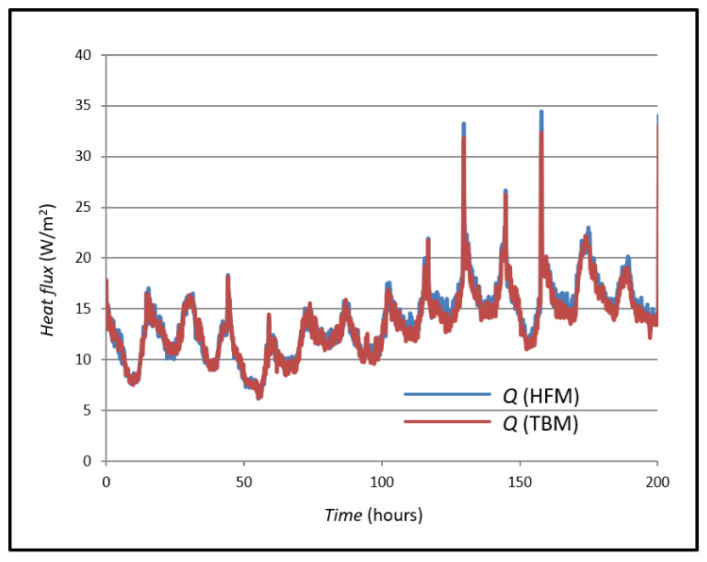
Measured and simulated heat fluxes in case A.

The thermal properties of the wall are determined by comparing the measured and calculated heat flux at the inner edge of the wall (x = 0). This approach is chosen because external weather variations significantly affect the heat flux at the outer edge (x = L). It is assumed that the heat flux measured at the inner edge by the sensor equals the heat flux entering the wall. Thermal conductivity (k) and thermal diffusivity (K) were iteratively estimated using the LABWIEV program. Initial values were adjusted until the simulated heat flux closely matched the measured values. Since temperature distribution and heat flux depend solely on these two thermal parameters, accurate results can be achieved if boundary and initial conditions are known. The comparison between simulated and measured heat flux is illustrated in Figure 9.

Figure 8 shows the *Q* value, its measured value with a heat flux sensor using the HFM method and the *Q* value of the heat flux measured and calculated using the TBM. The mean value of the heat flux using the TBM is 13.89 W/m^2^, and the mean value of the heat flux using the HFM method is 17.43 W/m^2^. The values were compared, and their mean difference was calculated as 3.54 W/m^2^.

In the first case, measured heat flux values were used to obtain the *U*-value according to Formula (3) as follows:

(3) $$\;U\;\left(\frac{W}{m^{2}K}\right)=\frac{Q}{T_{in}-T_{out}}=\frac{(Q_{1}+Q_{2})/2}{{(T}_{in1}+T_{in2}{)/2-(T}_{out1}+T_{out2})/2}\;$$

The results obtained were compared with the results obtained using the TBM model using Formula (4). *R_si_* is estimated using standard values for building materials and is 0.13 m^2^K/W [3]. In this case, the heat flux *Q* is obtained from the following formula:

(4) $$\;Q\;\left(\frac{W}{m^{2}}\right)=\frac{\left(T_{in}-T_{wall}\right)}{R_{si}}=\frac{(T_{in1}+T_{in2})/2-(T_{wall1}+T_{wall2})/2}{0.13}$$

The measured values of the *U*-value according to Formulas (3) and (4) are shown in Figure 9.

**Figure 10:**
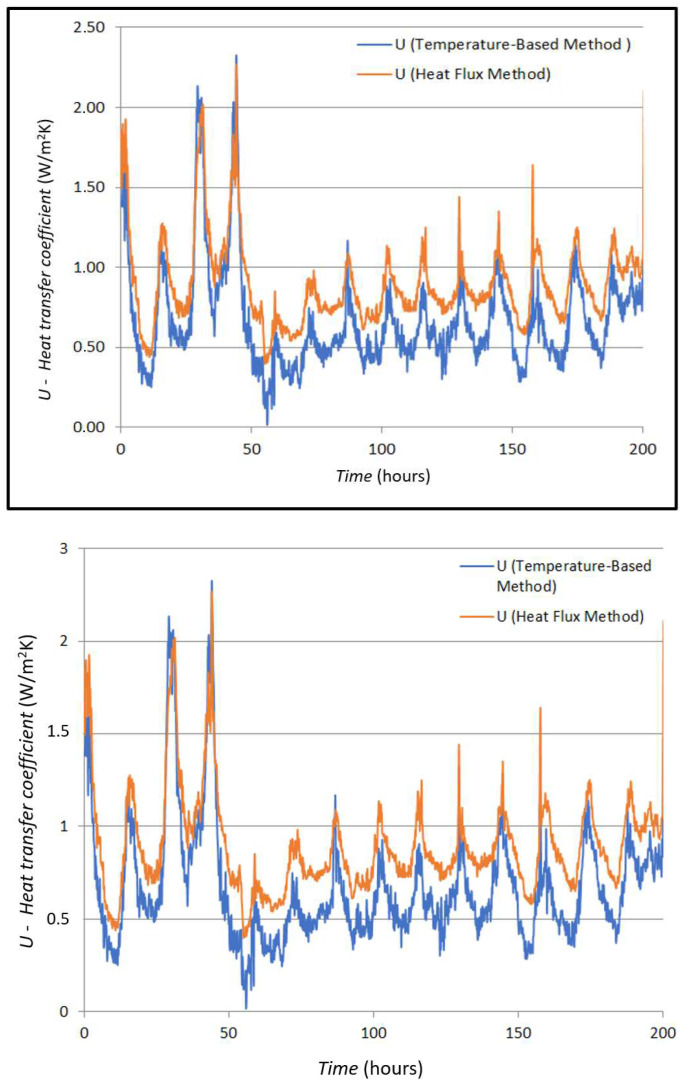
Comparison of measured and simulated *U*-values in case A.

The curves in Figure 10 show the mean value for the *U*-value measured by the HFM method (0.896 W/m^2^K) and the mean value of the *U*-value curve obtained by the TBM (0.673 W/m^2^K). If these two mean values are compared, a difference of 0.223 W/m^2^K is seen, which a difference of 24.88 percent.

In this case, the total measurement uncertainty of the HFM method is 1.86 W/m^2^. The standard deviation of the mean is 0.17 W/m^2^. This is uncertainty A. Uncertainty B is 0.02 W/m^2^. The total uncertainty is the root of the sum of the squares of A and B and is 0.18 W/m^2^, which is 7.12%.

The measurement uncertainty depends on the difference between the two temperatures *T_in_* and *T_wall_* and on the measurement uncertainty of the thermistors themselves, which is 30 mK.

The total measurement uncertainty of the temperature difference Δ *T* is 0.15 °C, the measurement uncertainty of the sensor is 0.04 °C, and the total measurement uncertainty is 0.06 °C, and the relative total uncertainty of the TBM method is 0.59%.

If the components of the wall structure are known and the thermal conductivity of the insulation materials is known as well, the *U*-factor can be estimated by calculating the total thermal resistance of all layers in the structure using the following equation:

(5) $$R=R_{EE}+\frac{d_{1}}{U_{1}}+\frac{d_{2}}{U_{2}}+\ldots +\frac{d_{N}}{U_{N}}+R_{IE}=\frac{1}{U}$$

Here, *R_EE_* represents the thermal resistance of the external environment; *U*_1_, *U*_2_, …, *U_N_* are the heat transfer coefficients of the individual material layers in the wall; *R*_IE_ is the thermal resistance of the internal environment; and *d* is the thickness of each individual layer [28].

**Figure 11:**
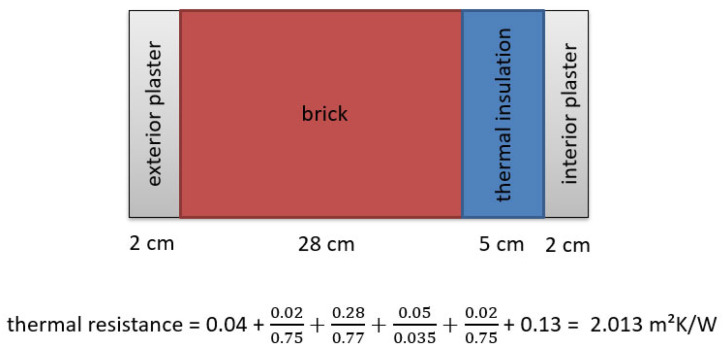
Wall construction and thermal resistance calculation.

First, the dimensions of each individual layer of material are determined, and their reciprocal thermal conductivity values are associated with them. The *U*-values are determined using the formula shown in Figure 11:

The thermal resistance values for different wall components are as follows: 0.04 m^2^K/W for external air, 0.75 m^2^K/W for external and internal plaster, 0.77 m^2^K/W for brick, 0.035 m^2^K/W for thermal insulation, and 0.13 m^2^K/W for internal air. This results in a total thermal resistance of 2.013 m^2^K/W, from which the *U*-value is calculated as 0.497 W/m^2^K. This value is lower than both the TBM and HFM method results; however, this calculation is only an approximation. Since the building was constructed in 1963, precise data on the actual wall composition are unavailable, and environmental or aging factors may further influence its thermal properties. Consequently, relying solely on calculations is inadequate for determining a building’s *U*-value; direct measurements are essential for accurate assessment.

In some measurement cases, significant differences arise between TBM (temperature-based method) and HFM (heat flux meter) method results, particularly in thicker walls. These discrepancies occur due to transient heat storage effects, which TBM does not account for but the HFM method detects, leading to deviations. Additionally, boundary conditions influence the HFM method more than the TBM, yet the TBM’s fixed assumptions may not align with real-world conditions. The HFM requires longer measurement periods for thick walls to capture thermal behavior accurately, whereas TBM often oversimplifies the process. Furthermore, the TBM’s assumed thermal resistance values may not reflect actual conditions, introducing systematic errors. Thus, the choice of methodology significantly impacts measurement accuracy. For thick walls, the most reliable approach is to make long-term HFM measurements supplemented by cross-checking TBM results with real boundary conditions to enhance accuracy.

### 3.2. Measurement of U_C_-Value of Window: Case B

The *U*-value of a window is a measure of its overall energy efficiency, encompassing the complete window assembly, which includes the glazing, frame, and spacer. The spacer, a component of the window frame that separates the glazing panels, plays a crucial role in reducing the *U*-value, particularly at the edges of the glazing.

In contrast, the performance rating specific to the glazing alone, independent of the frame, is referred to as the center-of-glass *U*-value. However, this rating is less commonly used in practice. For most energy-efficient windows, the *U*-value for the entire assembly tends to be higher than the center-of-glass *U*-value, reflecting the combined influence of the frame and spacer on the window’s thermal performance.

For case B, measurements were conducted in March when outdoor temperatures consistently remained below 10 °C. The interior was heated to approximately 20 °C, ensuring a temperature difference exceeding 10 °C, as required for the study. The same measurement system and methodology used in case A were applied to maintain consistency and reliability in the results.

**Figure 12:**
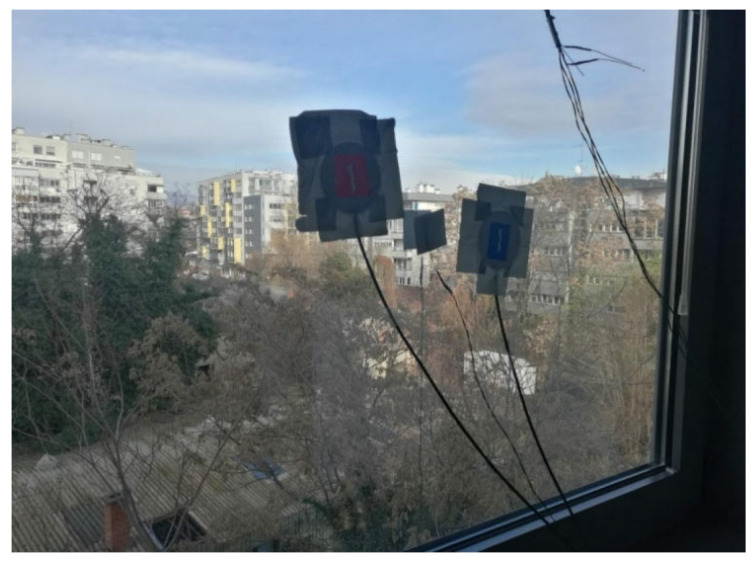
The window through which the heat flow was measured.

The *U*-value for most double-glazed windows is around 2.8 W/(m^2^K) [29], although this can vary depending on the manufacturer. This number refers to the rate of heat transfer through the window. The lower the *U*-value, the better the insulation and energy efficiency of the window. See Figure 12.

**Figure 13:**
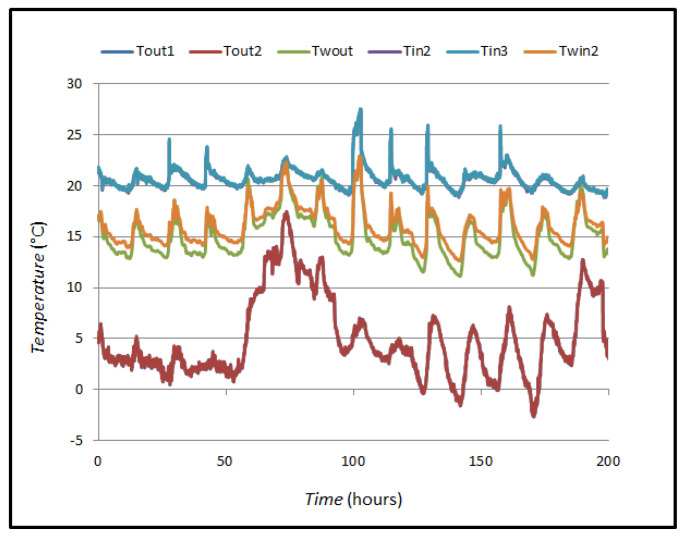
Measured temperature values on the outside of the window at 10 cm from the surface.

Each temperature value was measured with two sensors from which the mean value was taken. Figure 13 shows the measured temperature values on the outside of the window at 10 cm from the surface (*T_out_*_1_ and *T_out_*_2_), the measured temperature values on the glass itself on the outside (*T_wout_*), the measured temperature values on the inside of the window at 10 cm from the surface (*T_in_*_2_ and *T_in_*_3_), and the measured temperature values on the glass itself on the inside.

**Figure 14:**
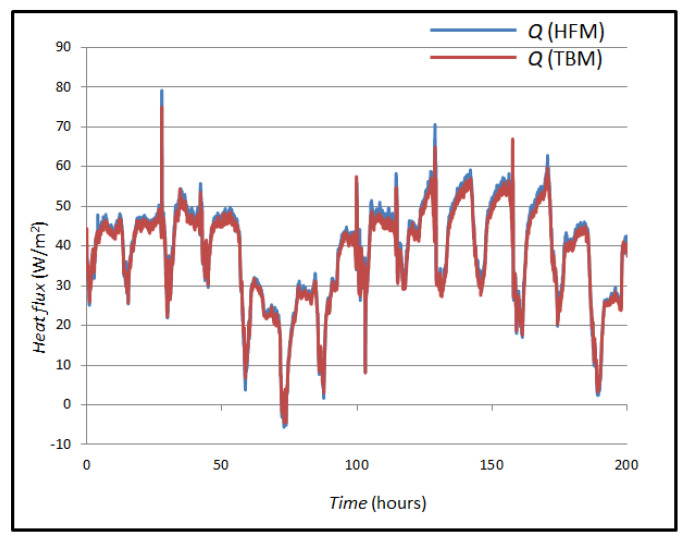
Measured heat flows in case B.

Figure 14 shows the *Q* value, its measured value with a heat flux sensor using the HFM method and the *Q* value of the heat flux measured and calculated using the TBM. The mean value of the heat flux using the HFM method is 38.64 W/m^2^, and the mean value of the heat flux using the TBM is 37.70 W/m^2^. The values were compared, and their mean difference was calculated, which is 0.94 W/m^2^.

**Figure 15:**
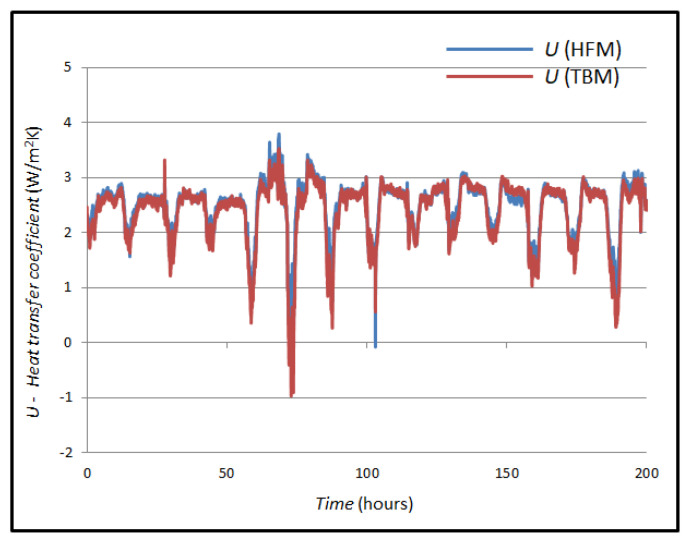
Comparison of *U*-values obtained by different measurement methods in case B.

Figure 15 shows the comparison between the calculated *U*-values in the TBM and HFM method. The mean value of the U-value using the HFM method is 2.401 W/m^2^K, and the mean value of the heat flux using the TBM is 2.485 W/m^2^K. The values were compared, and their mean difference was calculated, which is 0.084 W/m^2^K (3.38%).

If the measured *U*-values are compared with the theoretical values, a significant match is seen, since we do not have exact values for our window, and the *U*-value for most double-glazed windows is around 2.8 W/m^2^K.

In this case, the total measurement uncertainty of the HFM method is 1.2 W/m^2^. The standard deviation of the mean is 0.1 W/m^2^. This is uncertainty A. Uncertainty B is 0.02 W/m^2^. The total uncertainty is the root of the sum of the squares of A and B and is 0.12 W/m^2^ (4.64%). The total measurement uncertainty of the temperature difference Δ *T* is 0.03 °C, the measurement uncertainty of the sensor is 0.04 °C, the total measurement uncertainty is 0.05 °C, and the relative total uncertainty of the TBM is 0.11%.

Exact specifications are available for glass, while for the wall, they are assumed because we do not know the structure and aging effects of the wall. It should be emphasized that the measurement data are accurate, and these theoretical values are given for informational purposes only.

This study improves the clarity of measurement accuracy and methodology. The experimental setup supports the simultaneous application of the temperature-based method (TBM) and the heat flux meter (HFM) method, using a system equipped with 16 channels for temperature and heat flux sensors. This configuration allows for a real-time comparison of the two methods and the collection of a large amount of measurement data. To address the limitations of changing conditions, ISO 9869-1:2014 [1] recommends the use of an average method for determining the U-value. This approach involves analyzing measurements over time and averaging the data over several days. The device’s ability to record thousands of data points every 15 min, for each sensor, ensures a robust data set. During the analysis, outliers (such as events caused by people entering the room, opening windows or doors, or sudden changes in heating) were identified and excluded.

After filtering out these anomalies, the remaining data set was reviewed to identify periods of thermal stability, specifically times when both indoor and outdoor temperatures remained relatively constant. These stable intervals, which often occur at night due to minimized external disturbances, were selected for final averaging.

## 4. Conclusions

This study applied the modified heat flux meter (HFM) method to two cases: an external wall and a window of a measurement laboratory. The results were compared with the theoretical *U*-values derived from ISO 6946:2017 [27] and those obtained using the modified temperature-based method (TBM) for both cases. Using this method, better sensitivity and accuracy were achieved because a 6.5-digit high-precision digital multimeter was used for the measurement. Furthermore, all temperature sensors were calibrated to ensure a precision of greater than 1 mK between sensors.

From the results obtained, we can conclude that the modified TBM exhibits smaller deviations, giving results much closer to the theoretical *U*-value. Namely, the deviation from the theoretical value was 26.15% for the TBM compared to 44.53% for the HFM method. However, the theoretical *U*-value of the wall was calculated from incomplete data on the composition and construction of the wall itself. However, in the second case, when measuring the thermal transmittance of the window, the difference between the two methods is much smaller 3.38%.

The modified TBM produces results that are closely in line with the HFM method, but with the added advantage of significantly lower costs due to reduced dependence on expensive thermal flux sensors and measurement equipment needed to measure low value of voltage output of such sensors. Furthermore, the modified TBM showed greater agreement with the HFM method compared to theoretical U-value calculations, making it a cost-effective and reliable alternative for thermal transmittance assessment. By applying the modified TBM, building performance assessments can be conducted efficiently, providing essential insights for retrofitting and upgrading insulation without incurring excessive costs. The ability to conduct reliable evaluations with minimal financial investment makes this method particularly valuable when dealing with building and institutional complexes (e.g., hospitals, university campuses, military bases, etc.).

## Data Availability

Data is available upon request.

## Abbreviations

The following abbreviations are used in this manuscript:

HFM	Heat flux meter
TBM	Temperature-based method
T	Temperature
U	Thermal transmittance
R	Thermal resistance
Q	Heat flux
